# Fentanyl’s Deadly Footprint: A New Framework for Predicting Overdose Hotspots

**DOI:** 10.7759/cureus.100756

**Published:** 2026-01-04

**Authors:** Deborah Okunola, Abdulazeez Alabi, Olajide Akinpeloye, Osayimwense Izinyon, Tope Amusa

**Affiliations:** 1 Mathematics and Statistics (Biostatistics), Georgia State University, Atlanta, USA; 2 Epidemiology and Medical Statistics, University of Ibadan, Ibadan, NGA; 3 Statistics, Western Michigan University Homer Stryker M.D. School of Medicine, Kalamazoo, USA

**Keywords:** fentanyl overdose, hotspot prediction, public health surveillance, spatiotemporal analysis, wastewater-based epidemiology

## Abstract

Illicitly manufactured fentanyl has become a major driver of fatal drug overdoses in the United States, with annual mortality approaching six figures. Spatial analyses consistently demonstrate that the overdose burden clusters within structurally vulnerable micro-areas rather than distributing uniformly across regions. A narrative review of US studies (2010-2025) synthesized evidence from mortality, emergency department, emergency medical services, wastewater, social media, and related data streams that examined small-area patterns or the prediction of fentanyl or synthetic opioid overdose. Evidence shows a strongly clustered fentanyl burden, with small-area mapping identifying persistent hotspots and Bayesian forecasting and nowcasting models providing short-horizon predictions. Emerging wastewater and social media indicators offer additional early warning capacity, especially where routine surveillance is sparse. However, external validation, calibration reporting, uncertainty characterization, and equity-sensitive performance metrics are uncommon. This review organizes these strands into a three-tier framework--mortality-only, EMS and emergency department, and multi-stream environments--that links mapping, forecasting, and spike detection to operational response while embedding explicit equity and governance safeguards.

## Introduction and background

Fentanyl, a high-potency synthetic opioid, now anchors the contemporary US drug overdose crisis. National reports document sustained growth in deaths involving synthetic opioids and identify illicitly manufactured fentanyl as the dominant substance in fatal events. In 2022, almost 110,000 Americans died from drug overdoses. Opioids, particularly synthetic fentanyl, caused the majority of these deaths [1]. Within this landscape, “overdose hotspots” denote small geographic areas where fatal and nonfatal overdose events cluster in space and time at rates far above surrounding communities, creating concentrated zones of preventable harm that invite targeted public-health action.

The concentration of fentanyl-related harms across US jurisdictions has become clearer through spatial epidemiology and spatiotemporal modeling. Urban analyses from Cook County, Illinois, and other metropolitan settings reveal tighter spatial clustering of fentanyl-involved deaths than non-fentanyl overdoses, with hotspots closely aligned with structural disadvantage, racialized segregation, and unstable housing [1,2]. State- and national-level investigations extend this picture, describing shifting belts of synthetic-opioid mortality and persistent small-area clusters even where aggregate overdose counts have recently declined [3]. Recent work has moved beyond static maps toward small-area forecasting and near-real-time estimation of fentanyl harms, using combinations of routine surveillance, wastewater samples, and digital traces as inputs [4-8]. Guidance from national organizations, including overdose-spike response frameworks built around the Overdose Detection Mapping Application Program (ODMAP) and Emergency Medical Services (EMS) data, signals a shift from passive surveillance toward predefined local response protocols [9,10].

The resulting evidence base is technically rich but operationally thin: most fentanyl hotspot tools have not been organized into a realistic playbook for health departments. As detailed in subsequent sections, many models lack external geographic validation, have limited calibration or uncertainty characterization, and rarely provide stratified performance across racial, ethnic, and rural-urban strata. Fragmentation across mortality, emergency department, emergency medical services, wastewater, and social media data streams leaves health departments with isolated pilots rather than a coherent decision architecture, while existing spike-response frameworks provide limited guidance on how to embed equity safeguards and governance rules around hotspot outputs. This narrative review assembles disparate strands of fentanyl hotspot prediction into a three-tier framework: mortality-only, EMS and emergency department, and multi-stream environments, and specifies how each tier can use mapping, short-horizon forecasting, and spike detection to guide concrete public-health actions under explicit equity constraints. The framework targets US health departments, emergency medical services systems, and clinical networks that need to align overdose surveillance capacity with practical response playbooks in communities carrying the heaviest fentanyl burden.

## Review

Methodology

The review addressed data-driven prediction of fentanyl overdose hotspots in the United States, encompassing descriptive mapping, small-area forecasting, near-real-time nowcasting, extended surveillance streams, and overdose-response frameworks. Searches targeted studies analyzing fentanyl or synthetic-opioid overdose burden with explicit spatial resolution or predictive aims.

Electronic searches covered PubMed/MEDLINE, Web of Science, and Scopus, supplemented by targeted Google Scholar queries and screening of US public health agency and professional organization websites for guidance on overdose surveillance and spike response. Concept-level search strings combined terms for fentanyl, synthetic opioids, and overdose with terms for prediction, forecasting, clustering, hotspots, spatial analysis, wastewater, social media, syndromic surveillance, and alert frameworks. The search window spanned 1 January 2010 to 25 November 2025, with an emphasis on publications from 2019 onward. Only English-language sources focusing on US settings were considered.

Inclusion criteria required opioid- or fentanyl-related outcomes; small-area or neighborhood-level analyses (for example, county, ZIP code, census tract, emergency medical services catchment) or clearly defined spatial aggregation; and either quantitative prediction/nowcasting or operational frameworks linking surveillance to response. Preference favored studies reporting performance metrics, calibration or validation procedures, or explicit evaluation of implementation. Exclusions removed abstracts, letters, commentaries, national trend reports without spatial resolution, and non-US studies unless methods directly informed US practice. Reference lists of key sources informed additional snowball searches.

Data extraction captured settings, populations, data streams, spatial and temporal scales, modeling approaches, performance metrics, uncertainty handling, and equity or governance features. Evidence organization followed a tiered structure (mortality-only, emergency medical services, emergency departments, and multi-stream environments) to support the assessment of equity and governance implications. The research utilized a narrative review approach. Due to the varied nature of the included literature, encompassing diverse data sources, outcome definitions, analytical methods, and geographic scales, it was not feasible to employ formal systematic review procedures or meta-analytic techniques.

Results

National Burden and Broad Spatial Concentration of Fentanyl-Involved Overdose

Across multiple surveillance platforms, fentanyl now dominates the US overdose landscape. Friedman et al. reported the involvement of fentanyl in at least 75% of adolescent overdose deaths, with adolescent hotspot counties defined as those with ≥20 overdose deaths during the study period and concentrated in western and some southeastern states [11]. At the state level, Lu et al. documented an increase in fentanyl-detected fatal overdoses in Connecticut from 4% of all opioid-detected deaths in 2012 to 82% in 2019, based on 2009-2019 medical examiner data analyzed with Bayesian space-time models [12].

Emergency department (ED) data show parallel growth in nonfatal events. A national analysis of 3,056 facilities in the National Syndromic Surveillance Program identified an average quarterly increase of 5.4% (95% CI 4.4-6.5) in suspected fentanyl-involved nonfatal overdose ED visits per 10,000 ED encounters between quarter 4 (2020) and quarter 3 (2023) [13]. The same analysis reported an accelerated increase of 8.7% per quarter (95% CI 7.9-10.1) from quarter 3 (2023) to quarter 1 (2024), followed by an average quarterly decline of 11.0% (95% CI −16.9 to −2.8).

Spatial smoothing of overdose death rates at the county level across the contiguous United States has revealed a shift from earlier concentration in Appalachian and northeastern counties toward a more diffuse pattern, with synthetic-opioid and psychostimulant overdose clusters increasingly identified in parts of the Midwest and West during 2012-2020 [14]. These national and regional series position fentanyl-involved overdose as a dominant and geographically heterogeneous driver of current US overdose mortality and ED burden.

City- and State-Level Fentanyl Hotspot Patterns

Detailed city- and state-level analyses provide direct evidence of fentanyl-specific hotspots. In Cook County, Illinois, Nesoff et al. compared 1,433 fentanyl-involved fatal overdoses with 1,838 non-fentanyl and polydrug fatal overdoses from 2014-2018 using spatial point-pattern methods [2]. Fentanyl-involved deaths showed significantly stronger geographic clustering than non-fentanyl deaths, with a statistically significant high-risk area covering two Chicago neighborhoods [2]. Logistic regression in the same study estimated an odds ratio of 1.11 (95% CI 1.07-1.17) for fentanyl involvement per unit increase in a composite neighborhood deprivation score, after adjustment for individual-level demographics, indicating a concentration of fentanyl deaths in resource-deprived areas [2].

Kang et al. extended the spatiotemporal analysis of fentanyl-associated deaths in Chicago using ZIP-code-level mortality data from the Cook County Medical Examiner linked with American Community Survey covariates for 2018-2023 [15]. Global and local clustering statistics demonstrated stronger spatial autocorrelation during 2020-2021 and 2022-2023 than in 2018-2019, with high death rates initially concentrated in downtown Chicago and then expanding into surrounding ZIP codes over later periods [15]. Geographically weighted Poisson regression associated higher fentanyl death rates with larger local proportions of residents living in poverty, with disability, and in young-adult age bands; areas with higher educational attainment showed lower rates in parts of the city [15].

In Connecticut, Lu et al. used Bayesian space-time models to estimate the proportion of opioid-detected fatalities that involved fentanyl across towns between 2009 and 2019 [12]. Spatial random-effects surfaces indicated an early concentration of fentanyl-detected deaths in urbanized corridors, with later diffusion into additional towns, while the statewide proportion of fentanyl-detected deaths rose sharply during the same period [12].

State-level analyses using broader opioid endpoints capture additional context for hotspot prediction. Acharya et al. evaluated county-level emergency-department opioid-overdose visit rates in Virginia from 2016 to 2021, identifying persistent hotspots in several southwestern counties and persistent cold spots in many northern counties through local Moran’s I and LISA statistics [16]. A subsequent multilevel model associated higher ED overdose visit rates with worse clinical-care metrics and more adverse social and economic indicators at the county level, consistent with the concentration of overdose burden in structurally disadvantaged counties [16].

Hotspot Identification Using EMS and Near-Real-Time Administrative Data

EMS data offer finer temporal resolution for hotspot detection. Pesarsick et al. examined EMS runs for suspected opioid overdose and naloxone administration in a US jurisdiction, mapping incident locations to census tracts and applying spatial cluster detection [17]. The analysis pinpointed tracts exhibiting markedly increased ratios of overdose-related incidents in relation to population size, with high-risk clusters coinciding with regions of concentrated disadvantage [17].

Casillas et al. analyzed patient-level and county-level trends in nonfatal opioid-involved EMS encounters across 491 US counties between January 2018 and March 2022 [18]. That study documented a sustained increase in EMS encounters over time, with substantial inter-county variation. After adjusting for population, some counties had encounter rates several times higher than others [18].

Beyond individual studies, operational data systems such as the ODMAP aggregate incident-level reports from law enforcement and EMS agencies in near real time, enabling the identification of rapid local spikes in suspected overdose events at county or sub-county scales [10]. ODMAP implementation reports describe detection of short-term spikes and micro-hotspots over periods of days to weeks, based on sudden increases in recorded overdose events relative to recent baselines [19].

Small-Area Forecasting and Risk-Prediction Models

Several studies have moved from retrospective hotspot description toward a prediction of future high-risk areas. Bauer et al. developed Bayesian hierarchical models to forecast opioid-related mortality at the ZIP-code level in Massachusetts using mortality data from 2005-2019 and county-level covariates, generating predictions for 2020-2021 [4]. Model evaluation demonstrated good agreement between predicted and observed mortality rates, and forecasts identified ZIP codes projected to fall in the upper tail of the mortality distribution, providing a template for anticipatory identification of overdose hotspots [4].

A methodological review by Marks et al. synthesized prediction models for fatal and nonfatal opioid overdose events, including logistic regression, machine-learning classifiers, and survival models based on electronic health records, claims, and prescription-monitoring data [20]. Most models in that review lacked explicit spatial dependence, and external geographic validation rarely occurred, indicating limited translation of individual-level risk prediction into small-area hotspot forecasting [20].

At the county level, Ebrahimi et al. modeled county-level overdose mortality as a function of social determinants across the United States, treating fentanyl-associated mortality in Ohio as a key example [21]. Time-series and spatiotemporal models linked higher psychostimulant and cocaine overdose death rates to the presence of fentanyl in the local drug supply, with clusters of elevated risk in southern Ohio counties [22]. Related work on fentanyl-related overdose in Marion County, Indiana, used multilevel Bayesian models to estimate rapid growth in the proportion of overdose deaths involving fentanyl, with local rates approaching 50% of opioid deaths by 2017 [23].

Friedman et al. identified hotspot counties for adolescent overdose deaths in the adolescent population by exceeding certain rate thresholds and case counts. This led to the creation of a classification at the county level, which can serve as a basis for future predictive models [11]. Table 1 synthesizes design features, spatial scales, data sources, and headline estimates from key hotspot and forecasting studies.

**Table 1:** Core empirical studies informing fentanyl and opioid overdose hotspot prediction and surveillance The table summarizes eight core U.S. studies on opioid and fentanyl overdose hotspots, forecasting, and novel surveillance streams. Each row lists design, setting, comparators, outcomes, key findings, time window, and main limitations, illustrating how spatial clustering and predictive tools inform the tiered hotspot framework. SARIMA: Seasonal Autoregressive Integrated Moving Average; RMSE: Root Mean Squared Error

Study (author, year)	Design	Population/setting	Comparator	Outcome & metric	Headline estimate / main finding	Follow-up/window	Notable limits
Nesoff et al., 2020 [2]	Spatial analysis of medical examiner deaths; logistic regression	Cook County, Illinois; census tracts	Fentanyl-involved vs other opioid/polydrug deaths; deprivation gradient	Spatial clusters; odds of fentanyl involvement	High-deprivation tracts formed hotspots; deprivation associated with higher fentanyl odds	2014–2018	Single county; mortality only; no formal prediction
Bauer et al., 2023 [4]	Bayesian spatiotemporal dynamic model; small-area forecasting	Massachusetts; 537 ZIP Code Tabulation Areas	Models with vs without spatial/temporal dependence, covariates	One-year-ahead opioid-related deaths at the ZIP level	Dynamic spatial–temporal model best discriminated high- vs lower-risk ZIP codes	2005–2019 (forecast to 2020–2021)	Single state; all opioids; no external geographic validation
Sumner et al., 2022 [5]	National near–real-time nowcasting using multiple proxies	United States; weekly opioid-overdose deaths	Multi-proxy model vs SARIMA baseline	Weekly deaths; annual error, RMSE	Annual error ≈1% for 2018–2019; lower weekly RMSE than SARIMA	Training 2014–2017; validation 2018–2019	National scale only; no subnational hotspot outputs; not fentanyl-isolated
Endo et al., 2020 [6]	Wastewater-based opioid exposure and treatment assessment	U.S. city; 10 residential sewersheds	Higher vs lower metabolite loads in catchments	Overdoses and naloxone/buprenorphine loads in wastewater	Strong correlations between wastewater markers and local overdose/treatment indicators	Short pilot period	Single city; small N; correlation only; sewered areas only
Cuomo et al., 2023 [7]	Ecological prediction using opioid-related Twitter data	U.S. counties; mortality + Twitter posts	Models with Twitter features vs demographic/history baselines	County-level opioid-overdose mortality	Twitter-augmented models improved cross-county fit and captured temporal shifts	Multi-year county panel	Dependent on Twitter use; English-language bias; no prospective deployment
Lu et al., 2023 [12]	Ecological spatial–temporal analysis of fentanyl-detected deaths	Connecticut; state-wide mortality data	Fentanyl-detected vs non-fentanyl deaths; regions and years	Fentanyl-detected death rates by county and year	Rapid growth and shifting belt of fentanyl deaths across the state	2009–2019	One state; deaths only; polysubstance detail limited
Saunders, 2023 [14]	National spatiotemporal smoothing of overdose death rates	U.S. counties; three smoothed rate types	Persistently high vs lower-rate counties	Smoothed overdose death rates per 100,000	Persistent pockets of elevated overdose mortality, not a simple high–low gradient	2012–2020	Not fentanyl-specific; smoothing may mask micro-hotspots; no prediction
Kang et al., 2025 [15]	Spatiotemporal mortality analysis; ZIP-code level	Chicago, Illinois; fentanyl-associated deaths	Early vs later periods; high- vs lower-rate ZIP codes	ZIP-level death rates; hotspot persistence	Early ZIP hotspots persisted across periods with a gradual outward spread	2018–2023	Single city; descriptive; forecast accuracy not quantified

Novel Data Streams for Hotspot Prediction

Wastewater-based epidemiology has emerged as a candidate early-warning data stream. Endo et al. conducted an upstream wastewater-based monitoring pilot in a municipality in North Carolina, examining wastewater samples from 10 residential catchments for opioid metabolites, buprenorphine, and naloxone, and juxtaposing catchment-level detection rates with local opioid overdose rates and estimated naloxone dosages. The study reported a significant positive correlation between buprenorphine exposure and opioid overdose rate (Spearman ρ = 0.79, p = 0.028) and a weaker correlation for naloxone exposure (ρ = 0.58, p = 0.080), illustrating alignment between wastewater-derived opioid and naloxone indicators and local overdose burden at the community level [6].

Social media data have also been used to estimate overdose burden. Cuomo et al. built county-level models using Twitter-derived features combined with demographic covariates to predict opioid overdose mortality, demonstrating that models including Twitter signals explained a substantial proportion of cross-county variation in overdose death rates and captured temporal shifts in burden [7].

**Figure 1:**
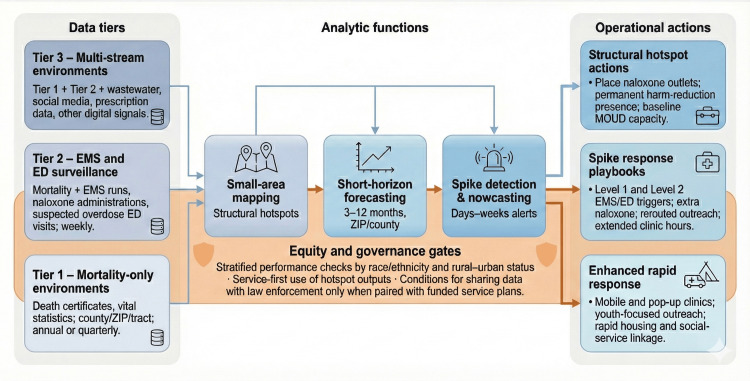
Tiered fentanyl overdose hotspot prediction framework by surveillance capacity The conceptual diagram illustrates the three data tiers (mortality-only, EMS and emergency department, and multi-stream) that feed analytic modules for small-area mapping, short-horizon forecasting, and spike detection. Outputs pass through equity and governance gates before triggering structural hotspot actions, spike response playbooks, and enhanced rapid response options. EMS: Emergency Medical Services; ED: Emergency Department; MOUD: Medications for Opioid Use Disorder Image credits: Tope Amusa

Incorporating non-traditional data streams, such as wastewater and social media studies, is feasible for use in hotspot-prediction frameworks. These studies are particularly valuable for short-term forecasting and for regions with limited traditional surveillance coverage, as demonstrated by their combined findings [6,7]. Figure 1 presents a multi-tiered framework for predicting fentanyl overdose hotspots. This framework organizes the synthesized evidence according to surveillance capacity, linking together data tiers, analytical functions, equity and governance checkpoints, and resulting operational actions.

Discussion 

Fentanyl hotspot prediction emerges from the assembled evidence as a useful instrument only under specific conditions. Reliable benefit appears when surveillance captures overdose events with adequate spatial and temporal resolution, models align with local data capacity rather than aspirational ideals, and equity checks operate explicitly so that hotspot labels trigger services instead of intensifying punishment. The absence of the stated conditions converts prediction into the relabeling of structural harm already visible in descriptive maps.

From Descriptive Mapping to Predictive Risk

Spatial analyses of fentanyl-involved and opioid-related deaths describe a sharply patterned geography rather than a uniform national burden. In Cook County, Illinois, Nesoff et al. mapped a bifurcated market in which fentanyl-involved deaths clustered tightly in neighborhoods with high deprivation indices, while non-fentanyl overdoses appeared more diffuse across the county [2]. Subsequent work in the same region tracked fentanyl-associated deaths between 2018 and 2023 and reported the persistence of early ZIP-code hotspots inside Chicago across successive four-year periods, with only gradual outward expansion [14,24]. Massachusetts analyses at the ZIP-code level reported smoothed opioid overdose death rates that formed pockets of persistently elevated risk, rather than a simple gradient from high- to low-burden counties [4].

Descriptive mapping at this resolution already reshapes operational decisions. Identification of persistent ZIP-code or tract-level hotspots supports alignment of naloxone distribution, outreach staffing, and low-barrier treatment capacity with local burdens rather than with county averages or political boundaries. Latency in the underlying data constrains that value. Death certificates and medical examiner results in many states lag by months; data on emergency department discharges often lag by weeks [25]. Maps constructed from such sources frequently portray risk from a prior season instead of risk over the coming months.

Forecasting and nowcasting methods reduce this delay. The Bayesian spatiotemporal model developed by Bauer et al. for Massachusetts used 15 years of mortality data across 537 ZIP Code Tabulation Areas and produced one-year-ahead forecasts of opioid-related death rates, with meaningful discrimination between upper-tail and lower-risk areas [4]. Temporal dependence and area-level covariates provided a sufficient signal to anticipate which localities would maintain or intensify high mortality. At a different scale, Sumner et al. combined emergency-department, law-enforcement, and online indicators to estimate national opioid-overdose deaths in near real time, achieving an annual error close to 1% for 2018-2019 and substantially lower weekly root-mean-square error than a standard SARIMA benchmark [5].

**Figure 2:**
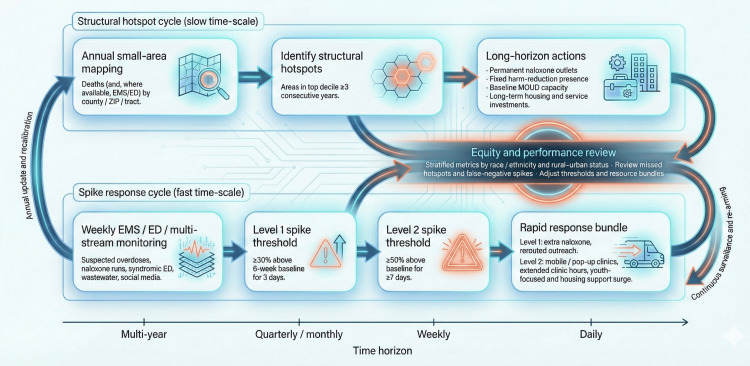
Hotspot-guided fentanyl overdose response cycle across structural and spike risk Diagram with two parallel tracks over a time horizon: the upper track shows an annual structural hotspot cycle (small-area mapping → hotspot designation → long-horizon actions); the lower track shows a fast spike-response cycle (weekly EMS/ED and multi-stream monitoring → Level 1 and Level 2 spike thresholds → rapid response bundle). Arrows from both tracks pass through an “Equity and performance review” node before looping back for updates and recalibration. EMS: Emergency Medical Services; ED: Emergency Department Image credits: Olajide Akinpeloye

Evidence from these strands supports a layered concept. Descriptive small-area maps identify locations of structurally entrenched overdose burden, often with substantial delay but with strong geographic resolution. Forecasting and nowcasting models add either a longer planning horizon (months to a year in Bayesian small-area models) or a shorter lag behind real-world events (weeks in nowcasting from proxy data). A credible fentanyl hotspot framework, therefore, treats the descriptive and predictive layers as complementary: descriptive mapping provides the baseline geography of risk, and prediction, along with nowcasting, adjusts that geography in time to inform response. However, these predictive gains remain context-dependent and may be constrained by data quality, model uncertainty, and uneven local surveillance capacity. Figure 2 sets out the hotspot-guided response cycle, aligning structural hotspot designation, predefined spike thresholds, and response bundles along multi-year to daily time horizons under a shared equity review gate.

Data-Tiering and Model Selection for Different Public-Health Capacities

The reviewed evidence undermines any claim that a single “best model” suits all jurisdictions. Data environments and analytic capacity vary widely, and the literature already suggests sensible pairings between context and method.

In data-sparse settings, mortality statistics and occasional emergency-department summaries may constitute the only robust signals. Massachusetts small-area forecasting work indicates that such a minimal stack can still support annual ZIP-code risk prediction when multiyear death data and basic demographic information are available [4]. Routine application of similar models would provide state health departments and local coalitions with a more stable view of structurally high-risk communities, even before incorporation of faster data streams.

Arrival of near-real-time EMS and syndromic emergency-department feeds alters the feasible strategy. EMS datasets used for spatial clustering and trend analysis, together with syndromic surveillance, support daily or weekly counts at fine spatial scales [26]. Threshold-based rules and simple anomaly-detection procedures can then trigger spike alerts without complex modeling. The National Association of County and City Health Officials (NACCHO) overdose-spike response framework already employs ODMAP data in this fashion, with alerts based on deviations from recent baselines over short windows [9,10]. Such systems improve timeliness substantially relative to mortality statistics, even when statistical machinery remains modest. The combined evidence indicates that this intermediate tier offers a pragmatic starting point for many counties and cities because EMS and emergency department pipelines, along with basic governance arrangements, already operate in those settings.

The current frontier is occupied by a multi-stream tier. Wastewater-based monitoring pilots in North Carolina and other jurisdictions report strong correlations between opioid metabolite loads and local overdose burden [6,8], and Twitter- or Reddit-based models capture additional variation in county-level overdose mortality beyond demographic covariates and historical deaths [7]. Integrated models that combine lagged mortality, real-time EMS and emergency-department data, wastewater indicators, and social-media signals can, in principle, improve spatial resolution and extend lead times [5-8]. External validation and robustness testing for multi-stream architectures remain limited, and published examples of sustained deployment within public health agencies are scarce [5,7,27]. Evidence, therefore, supports cautious recommendations: jurisdictions with sufficient infrastructure can pilot multi-stream models but should retain simpler, interpretable baselines for operational decisions until comparative performance, maintenance demands, and failure modes become clear.

Across all tiers, predictive modeling must address uncertainty arising from under-ascertainment, misclassification of fentanyl involvement, and evolving drug markets. Reviewed studies rarely report calibration metrics, confidence intervals for risk rankings, or sensitivity analyses under alternative assumptions about data completeness. Framework design benefits from explicit presentation of uncertainty and conservative action thresholds, particularly when hotspot labels may trigger intensive interventions [20].

Equity, Ethics, and Unintended Consequences of Hotspot Prediction

Spatial analyses in Chicago, Connecticut, Massachusetts, Virginia, and other regions repeatedly link higher fentanyl or opioid overdose rates to neighborhood deprivation, unstable housing, and limited access to healthcare [2,21,28,29]. Adolescent-focused work reports similar concentration: fentanyl involvement appears in at least three-quarters of adolescent overdose deaths in recent national analyses, and hotspot counties exhibit intersecting vulnerabilities such as counterfeit pill exposure and prior mental health conditions [11,30,31]. Such findings imply that hotspot prediction will mark communities already subject to social and economic marginalization.

Concentration in deprived areas generates ethical tension. Predictive labels can guide harm-reduction investments such as high-coverage naloxone distribution, youth-oriented outreach, and low-barrier treatment entry. The same labels can also be used to justify more police activity and move drug use out of sight, without adding any more services. Regional data analyses and investigative reporting document periods where national overdose counts plateau or decline while deaths rise in specific states and counties with persistent racial and socioeconomic disparities [25,28]. Predictive frameworks that disregard governance context may exacerbate punitive responses rather than channeling resources toward treatment and social support.

Equity concerns extend into data sources and models. Twitter- and Reddit-based approaches depend on social media access and English-language posting patterns, conditions that underrepresent rural communities, older adults, and speakers of other languages [7]. Wastewater sampling frames frequently exclude unsewered rural areas and informal housing, biasing coverage toward residents connected to municipal sewer systems [6,8]. Most modeling studies do not report stratified accuracy metrics or false-negative rates for marginalized groups. Absent such reporting, hotspot frameworks risk lower sensitivity or poorer calibration in communities where overdose deaths increase fastest [4,7,32].

An ethically defensible hotspot framework embeds equity safeguards as core design elements. One safeguard should be a periodic review of hotspot maps and alert patterns against independent indicators of social disadvantage and demographic composition; disagreement between predicted risk and observed overdose trends in minority communities should trigger model diagnosis, retraining, or augmentation with alternative data sources. A second safeguard can require documentation that service expansion and harm-reduction measures constitute the primary response to hotspot alerts, with any law-enforcement use subject to separate scrutiny and community oversight. Placement of such checkpoints inside the operational pathway converts broad ethical concern into a concrete constraint on action.

Positioning the Framework Within the Overdose-Response Ecosystem

Guidance from NACCHO and allied organizations already outlines overdose-spike response frameworks that couple ODMAP or EMS data with predefined alert thresholds and playbooks for multi-agency mobilization [9,10,33]. Documents from ASTHO and federal partners similarly emphasize the formation of overdose spike response teams, action plans, and continuous quality improvement around EMS and law enforcement data streams [33].

The synthesis presented here supports extension of that architecture along three axes: predictive depth, spatial granularity, and equity governance. Predictive depth increases when mortality and emergency-department surveillance join EMS data, nowcasting models compensate for reporting lag, and Bayesian or other small-area forecasting methods identify neighborhoods at sustained risk. Spatial granularity improves when analyses move from state and county averages to ZIP codes, census tracts, and EMS catchments, as already demonstrated by several state and city studies. Equity governance strengthens when frameworks adopt explicit rules about acceptable uses of hotspot outputs and embed regular bias audits.

A fentanyl-focused public health response framework grounded in the reviewed evidence rests on three linked components. Baseline descriptive mapping based on the most complete mortality and emergency-department data defines persistent hotspots and supplies a scaffold for long-term intervention planning. Predictive modules tailored to local capacity, ranging from simple spike-detection algorithms on EMS feeds to multi-stream machine-learning models, supply short-term alerts and medium-term forecasts, with transparent documentation of uncertainty. Decision pathways derived from tools such as the NACCHO overdose-spike response framework then translate risk signals into packages of response actions that prioritize naloxone distribution, timely outreach, and treatment access, with required checks for disproportionate burden or under-detection in marginalized communities [9,10,33].

Current evidence still reveals several gaps that constrain such a framework. Prospective evaluations of hotspot-guided interventions remain rare, external validation for many predictive models is limited, and reporting standards for small-area overdose forecasting have not converged around common calibration and equity metrics [27]. Even with these limitations, the assembled data across surveillance modalities justify structured adoption of predictive hotspot tools within US fentanyl-response strategies, provided that surveillance quality, model choice, and equity safeguards occupy the center of framework design rather than the margins.

Implications for Public Health and Clinical Practice

Hotspot prediction only matters if it changes what public-health teams do on Monday morning. The evidence in this review points to a few concrete shifts in practice rather than a new layer of dashboards.

Every jurisdiction needs a baseline for managing itself: pick a minimum data tier and make it non-negotiable. For a typical state health department, that promise looks modest but concrete over a 12-18-month horizon: (a) link death certificates, ED overdose visits, and EMS runs at least to the ZIP-code level; (b) publish small-area overdose maps quarterly; and (c) run a simple one-year forecast that flags ZIP codes in the top decile of predicted fentanyl or opioid mortality. Any ZIP that sits in that top decile for three consecutive years, a proposed threshold informed by observed hotspot persistence patterns in the reviewed literature, should be treated as a structural hotspot, not a temporary spike: fixed naloxone outlets, permanent harm-reduction presence, and stable Medications for Opioid Use Disorder (MOUD) capacity become baseline obligations rather than pilot projects [15].

Spike alerts also need something more than dashboards: a script that everyone around the table has seen before the crisis hits. A county can decide, in advance, that 'Level 1' EMS spikes (illustratively, ≥30% above a 6-week baseline for 3 days) trigger extra naloxone shipments and re-routing of existing outreach, whereas 'Level 2' spikes (illustratively, ≥50% above baseline for 7 days or more) trigger additional mobile clinics, extended clinic hours, and rapid contact with housing and youth-service partners. These thresholds are illustrative; jurisdictions should calibrate cutoffs based on local baseline variability and response capacity. If complex multi-stream or machine-learning models exist, their alerts should be forced to compete with these simple rules in a side-by-side evaluation before taking over escalation decisions.

Third, equity gates cannot be optional. Before any hotspot map influences resource allocation, the health department should run a brief audit: overdose rates and coverage of naloxone and MOUD stratified by race, ethnicity, and rural-urban status in flagged areas versus unflagged ones. If predictions miss rising deaths in a marginalized community or systematically undercall the risk there, the framework should treat that as a model failure, not a community failure. Hotspot outputs should stay out of law enforcement workflows unless a governance group, ideally with community representation, signs off on a parallel, funded service plan for the same streets.

## Conclusions

Evidence across mortality, emergency department, emergency medical services, wastewater, and social media sources shows that fentanyl overdose risk concentrates in identifiable neighborhoods and counties rather than diffusing evenly across the United States. Tiered hotspot prediction, anchored in small-area mapping, short-horizon forecasting, and near-real-time spike detection, offers a practical route to earlier, more targeted public-health action when grounded in adequate surveillance and equity safeguards. Future progress depends on prospective evaluation of hotspot-guided interventions, rigorous external validation of predictive models, and adoption of reporting standards that incorporate calibration, transparency, and stratified performance across vulnerable populations.
